# Radiomic feature clusters and Prognostic Signatures specific for Lung and Head & Neck cancer

**DOI:** 10.1038/srep11044

**Published:** 2015-06-05

**Authors:** Chintan Parmar, Ralph T. H. Leijenaar, Patrick Grossmann, Emmanuel Rios Velazquez, Johan Bussink, Derek Rietveld, Michelle M. Rietbergen, Benjamin Haibe-Kains, Philippe Lambin, Hugo J.W.L. Aerts

**Affiliations:** 1 Departments of Radiation Oncology; 2 Radiology, Dana-Farber Cancer Institute, Brigham and Women’s Hospital, Harvard Medical School, Boston, MA, USA; 3 Radiation Oncology (MAASTRO), Research Institute GROW, Maastricht University, Maastricht, the Netherlands; 4 Machine Intelligence Unit, Indian Statistical Institute, Kolkata, India; 5 Department of Biostatistics & Computational Biology, Dana-Farber Cancer Institute, Boston, MA, USA; 6 Department of Radiation Oncology, Radboud University Medical Center, Nijmegen, the Netherlands; 7 Department of Radiation Oncology, VU University Medical Center, Amsterdam, The Netherlands; 8 Department of Otolaryngology/Head and Neck Surgery, VU University Medical Center, Amsterdam, The Netherlands; 9 Ontario Cancer Institute, Princess Margaret Cancer Centre, University Health Network, Toronto, Ontario, Canada; 10 Medical Biophysics Department, University of Toronto, Toronto, Ontario, Canada

## Abstract

Radiomics provides a comprehensive quantification of tumor phenotypes by extracting and mining large number of quantitative image features. To reduce the redundancy and compare the prognostic characteristics of radiomic features across cancer types, we investigated cancer-specific radiomic feature clusters in four independent Lung and Head & Neck (H∓N) cancer cohorts (in total 878 patients). Radiomic features were extracted from the pre-treatment computed tomography (CT) images. Consensus clustering resulted in eleven and thirteen stable radiomic feature clusters for Lung and H & N cancer, respectively. These clusters were validated in independent external validation cohorts using rand statistic (Lung RS = 0.92, p < 0.001, H & N RS = 0.92, p < 0.001). Our analysis indicated both common as well as cancer-specific clustering and clinical associations of radiomic features. Strongest associations with clinical parameters: Prognosis Lung CI = 0.60 ± 0.01, Prognosis H & N CI = 0.68 ± 0.01; Lung histology AUC = 0.56 ± 0.03, Lung stage AUC = 0.61 ± 0.01, H & N HPV AUC = 0.58 ± 0.03, H & N stage AUC = 0.77 ± 0.02. Full utilization of these cancer-specific characteristics of image features may further improve radiomic biomarkers, providing a non-invasive way of quantifying and monitoring tumor phenotypic characteristics in clinical practice.

Recent advances of medical and computational science have led to the emergence of ‘precision medicine’, which has revolutionized the cancer care and medical science in general. A major proportion of precision medicine research has centered on unveiling different molecular characteristics of the disease tissues by using genomic and proteomic technologies. In spite of their enormous potential, these techniques have found limited implementations in routine clinical practice^1^. A major challenge being the invasive nature, as biopsies, having high associated risk and cost, are often required.

Imaging on the other hand provides promising means of non-invasive tissue characterization and is furthermore routinely used for disease detection, diagnosis, and treatment purposes in clinical practice^2^,^3^,^4^. X-ray computed tomography (CT) is a frequently used imaging modality for oncology because it assesses tissue density in high resolution and exhibits strong contrasts among different tissue types. In routine clinical practice, tumor response to therapy is measured by the RECIST and/or WHO criteria, based on CT imaging. These descriptors measure the change in size of tumors, and often do not succeed in predicting overall survival^5^,^6^.

“Radiomics” is an emerging field of research that aims to utilize the full potential of medical imaging. Radiomics focuses on extracting a large number of quantitative features from medical images, providing a more detailed quantification of tumor phenotypic characteristics–effectively converting medical images into a high dimensional minable feature space^7^,^8^,^9^. Several studies have defined and quantified various image descriptors and stated their significance for treatment monitoring and outcome prediction in different cancer types^10^,^11^,^12^,^13^,^14^. Moreover, some studies have also reported an association between radiographic imaging phenotypes and tumor stage, metabolism^15^, hypoxia, angiogenesis^16^ and the underlying gene and/or protein expression profiles^17^,^18^,^19^.

A main challenge in radiomics is to deal with feature redundancy in order to obtain a non-redundant set of imaging biomarkers. Consensus clustering^20^ could address this issue by reducing the feature space into several non-redundant feature clusters. In this study we identified and validated radiomic feature clusters in cohorts of Lung cancer and Head & Neck (H∓N) cancer patients. We also evaluated the clinical importance of these clusters by quantifying their association with important clinical parameters and patient survival. Moreover, we used the identified radiomic clusters to build cancer-specific multivariable radiomic signatures and tested their prognostic performance. Identification of cancer-specific radiomic clusters provides a crucial step towards stable and clinically relevant radiomic biomarkers, providing a non-invasive way of quantifying and monitoring tumor phenotypic characteristics in clinical practice.

## Methods

### Radiomic features

We defined 440 radiomic image features that quantify tumor characteristics. These features were divided in four groups: I) tumor intensity, II) shape, III) texture and IV) wavelet features. Tumor intensity based features, which are defined using first order statistics of the intensity histogram, quantified the density of the tumor region on CT image. Shape features described the 3D geometric properties of the tumor, whereas textural features quantified intra-tumor heterogeneity. Textural features were computed by analyzing the spatial distribution of voxel intensities in thirteen directions. These features are derived from gray level co-occurrence (GLCM)^21^ and run length matrices (GLRLM)^22^ and were computed by averaging their values over all thirteen directions. Wavelet features are the transformed domain representations of the intensity and textural features. These features were computed on different wavelet decompositions of the original image using a coiflet wavelet transformation. All image analysis was performed in Matlab R2012b (The Mathworks, Natick, MA) using an adapted version of CERR (Computational Environment for Radiotherapy Research)^23^ and features were automatically extracted with in-house developed radiomics image analysis software. Mathematical definitions of all radiomic features as well as the extraction methods were previously described^18^.

### Datasets

**Figure 1:**
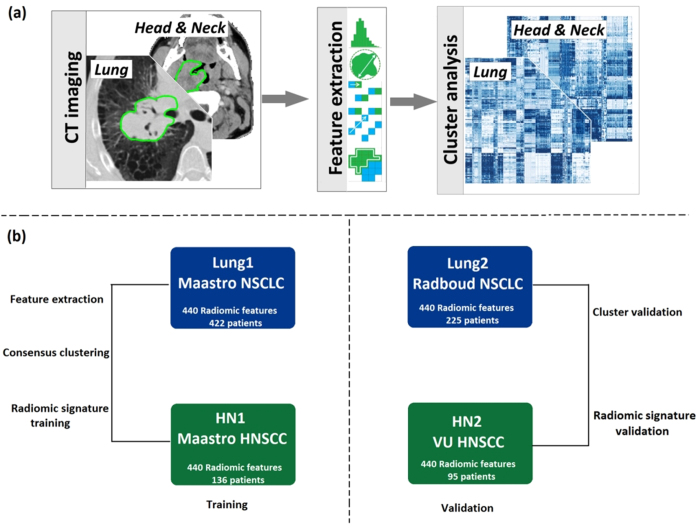
(**a**) Radiomic analysis overview: For both Lung and H & N cancer datasets, we extracted radiomic features from pre-treatment CT images. Cluster analysis was performed on the feature data. (**b**) Datasets overview: Four independent radiomic cohorts of Lung and Head & Neck cancer were included in the analysis. Lung1 and HN1 were used for training; Lung2 and HN2 were used for validation.

Briefly, we considered four image datasets (see overview in Figure 1) for this study, from different institutes in the Netherlands: (1) Lung1: 422 NSCLC patients treated at MAASTRO Clinic in Maastricht. (2) Lung2: 225 NSCLC patients treated at Radboud University Medical Center in Nijmegen. (3) HN1: 136 head and neck squamous cell carcinoma (HNSCC) patients treated at MAASTRO Clinic in Maastricht and (4) HN2: 95 HNSCC patients treated at the VU University Medical Center in Amsterdam. CT-scans, manual delineations and clinical data were available for all included patients. More details on the included datasets have been described earlier^18^.

### Data analysis

#### Comparison of the prognostic performance of radiomic features in Lung and H & N cancer

In order to compare the prognostic utility of radiomic features across Lung and H & N cancer, for each feature, we computed and compared the concordance index (CI)^24^, which is the generalization of area under ROC curve. R package survcomp was used for the analysis^25^. P-values are corrected for multiple testing (FDR 5%).

#### Consensus clustering

We used consensus clustering to cluster the radiomic features extracted from the training cohorts Lung1 and HN1. Consensus clustering is a resampling based clustering methodology, which quantifies the consensus between several clustering iterations and provides means to estimate the number of clusters that best fit the data^20^. We estimated the range for the appropriate number of clusters from the delta area plots (supplementary figure S1). From this range, we chose the number of clusters which gave the highest median cluster consensus over all clusters. Cluster consensus was defined as the average consensus between all pairs of features belonging to the same cluster. Cluster consensus (range [0–1]) indicates the robustness (stability) of a cluster over resampling. We also computed the mean pairwise correlation (range [0–1]) between features of a cluster, which is a measure of the cluster compactness (similarity of features within the cluster). Qualitative categorization of cluster stability was defined as; consensus < 0.5, poor stability; 0.5 ≤ consensus < 0.75, moderate stability; and consensus ≥ 0.75, high stability. Cluster compactness was also assessed using the same qualitative categorization. We applied hierarchical clustering with agglomerative ward linkage, a Pearson correlation based dissimilarity measure (1 − *r*) and 10,000 resampling iterations. Consensus clustering was performed using the R package ConsensusClusterPlus^26^.

#### Radiomic cluster validation

Radiomic feature clusters obtained on the training cohorts, Lung1 and HN1, were considered as the reference Lung and H & N clusters. For cluster validation, we clustered the Lung and H & N validation cohorts, Lung2 and HN2, using the same hierarchical clustering algorithm and the same number of clusters as for the corresponding training cohort. Rand Statistic (RS)^27^ was used to assess the agreement between each reference clustering (P) and the clustering obtained for its respective validation cohort (C) and was defined as:


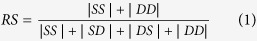


where *|SS|* is the number of feature pairs that cluster together in both *C* and *P, |SD|* is the number of feature pairs that cluster together in *C* but not in *P, |DS|* is the number of feature pairs that cluster together in P, but not in C and *|DD|* is the number of feature pairs that do not cluster together in both C and P. Significance of RS was determined by a random permutation test using 1000 iterations.

#### Similarity between Lung and H & N clusters

Cluster overlap between the individual feature clusters of Lung and H & N radiomic cohorts were assessed using the Jaccard index^27^, which is defined as:


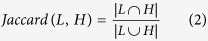


where *L* and *H* are any feature clusters of Lung and H & N cohorts. Qualitative categorization of cluster overlap was defined as; Jaccard < 0.5, poor overlap; 0.5 ≤ Jaccard < 0.75, moderate overlap; and Jaccard ≥ 0.75 high overlap.

#### Clinical relevance of radiomic clusters

In order to quantify the association between radiomic feature clusters and patient survival, we used the concordance index (CI), whereas the association between a feature cluster and a categorical clinical parameter (i.e. Lung cancer histology, H & N HPV status or Lung/H & N tumor stage) was quantified using the area under the ROC curve (AUC). For clinical parameters having more than two categorical levels, a multi-class AUC was computed using a pairwise approach. Univariable CI and AUC were computed for each feature. A cluster’s association with patient survival and clinical parameters was then quantified as the mean CI and mean AUC over all contained features. Significance was estimated using a random permutation test with 1000 iterations. R package survcomp^25^ and pROC^28^ was used for this analysis. Qualitative categorization of the prognostic or predictive performance was defined as poor (CI or AUC < 0.6), moderate (0.6 ≤ CI or AUC < 0.75) and high (CI or AUC ≥ 0.75).

#### Multivariable clinical relevance

In order to select non-redundant imaging biomarkers, cancer-specific radiomic signatures were built using the medoids of the obtained clusters of Lung1 and HN1 cohorts. The medoid is a single representative feature, which has the highest average pairwise correlation within a cluster. To investigate the multivariable prognostic utility of these selected radiomic features, a multivariable Cox proportional hazards model was fitted on each training cohort (i.e. Lung1 and HN1) and their prognostic performance was tested on validation cohorts (i.e. Lung2 and HN2) using the CI. R package survcomp^25^ was used for this analysis. For the prediction of categorical clinical parameters, we built multivariable classifiers on each training cohort (i.e. Lung1 or HN1) using logistic regression, with the medoids as independent variables. The predictive performance of a classifier was evaluated on the corresponding validation cohort (Lung2 or HN2) using AUC. For clinical parameters having more than two categorical levels, logistic regression was fitted using a pairwise approach and performance was evaluated using multiclass AUC^29^. R package VGAM^30^ was used for this analysis.

## Results

In order to investigate radiomic features in Lung and H∓N cancer cohorts, a total of 440 radiomic features were extracted from the segmented tumor regions of the pre-treatment CT images of Lung and Head and Neck cancer cohorts. In our analysis, we used datasets Lung1 (n = 422) and HN1 (n = 136) as training datasets, and Lung2 (n = 225) and HN2 (n = 95) as validation datasets (Fig. 1).

### Comparison of the prognostic performance of radiomic features in Lung and H∓N cancer

**Figure 2:**
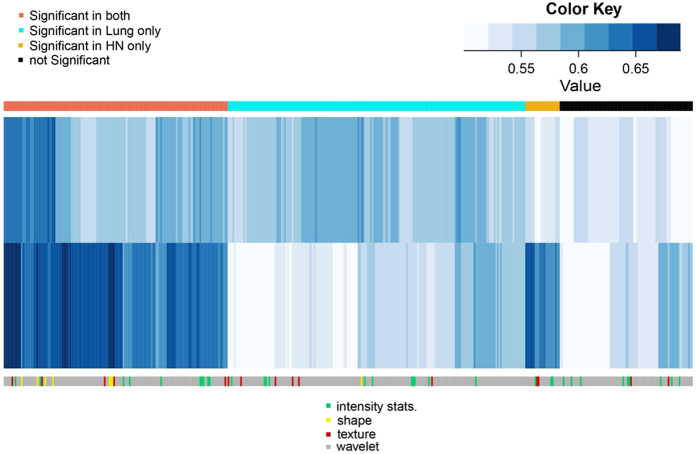
Heatmap showing the prognostic performance of radiomic features in Lung2 and HN2 cohorts. Prognostic performance was evaluated using the concordance index (CI). Note that a large number of features are prognostic in both cancer types. However, also a large number of features are cancer-type specific, e.g. prognostic only in Lung or only in H & N cancer.

The prognostic utility of the radiomic features was assessed using the concordance index (CI). Figure 2 depicts a CI heatmap of radiomic features in the validation cohorts (Lung2 and HN2). We observed that 143 features had significant prognostic performance (CI > 0.5, p < 0.05 FDR corrected) in both the cancer types whereas 212 features (190 features in Lung and 22 features in H∓N) showed significant prognosis only in one of the two cancer types. Eighty-five features turned out not to be significantly prognostic in either of the two cancer types.

### Identification and validation of radiomic feature clusters in Lung cancer

**Figure 3:**
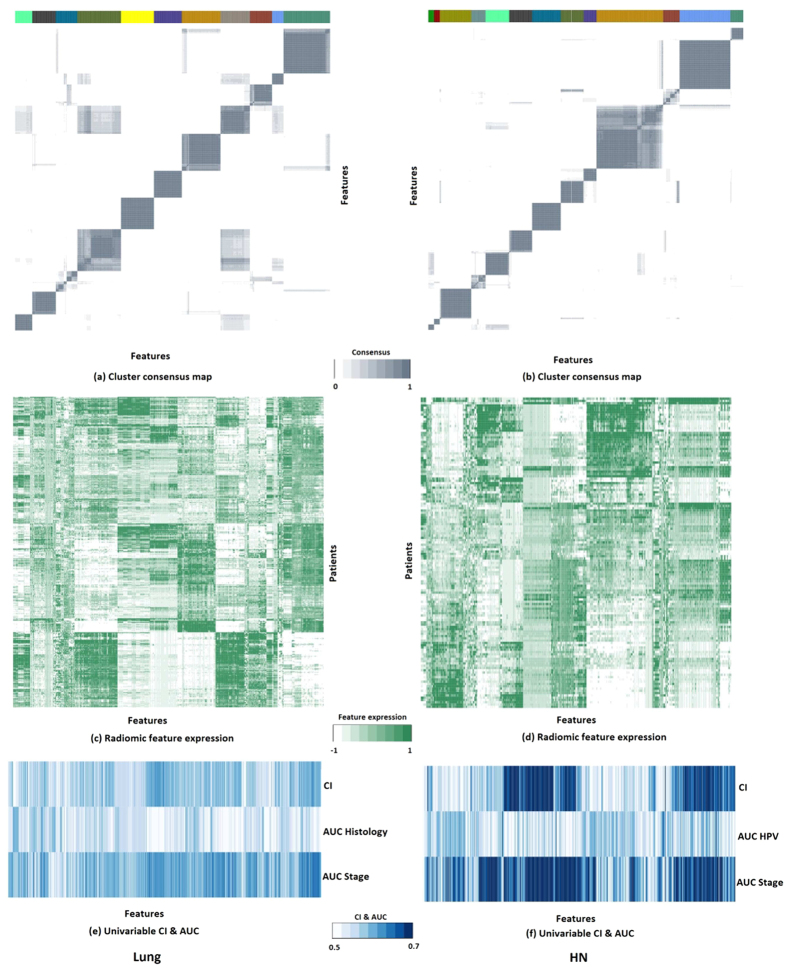
Heatmaps for radiomic features of Lung and H & N training cohorts ordered with respect to the obtained Lung and H & N clusters. (**a–b**) Cluster consensus maps of Lung cancer (11 clusters) and H & N cancer (13 clusters) cohorts. (**c–d**) Radiomic feature expressions of Lung and H & N radiomic clusters. (**e–f**) Clinical relevance (CI & AUC) of radiomic clusters of Lung and H & N cancer.

In order to identify stable clusters of radiomic features, consensus clustering procedure was applied on Lung1 training cohort. We obtained eleven distinct clusters (size: 16 to 65 features per cluster). Heatmaps in Figure 3 depict the consensus maps (Fig. 3a) and normalized expression levels (Fig. 3c) of the obtained Lung radiomic feature clusters in Lung1 training cohort. These clusters were validated in the Lung2 validation cohort (RS = 0.92, permutation test p-value < 0.001). We observed that four clusters (LCL-4, LCL-6, LCL-7, LCL-11) had a high cluster consensus (consensus ≥ 0.75) and within cluster correlation (correlation ≥ 0.75), whereas six clusters (LCL-1, LCL-2, LCL-3, LCL-5, LCL-8, LCL-10) showed high cluster consensus (consensus ≥ 0.75) but moderate within cluster correlation (0.5 ≤ correlation < 0.75). For cluster LCL-9, both the cluster consensus and within cluster correlation were poor (consensus = 0.41, correlation = 0.14). Details regarding the cluster size, associated feature categories, cluster consensus and within cluster correlation can be obtained from Table 1.

### Identification and validation of radiomic feature clusters in HN cancer

Consensus clustering in HN1 training cohort resulted in thirteen distinct radiomic feature clusters (size: 8 to 93 features per cluster), which were validated in independent HN2 validation cohort (RS = 0.92, permutation test p-value < 0.001). Heatmaps in Figure 3b and 3d show the consensus maps and normalized expression levels of the obtained radiomic feature clusters in HN1 training cohort. Six clusters (HNCL-1, HNCL-2, HNCL-6, HNCL-7, HNCL-12, HNCL-13) had high cluster consensus (consensus ≥ 0.75) and within cluster correlation (correlation ≥ 0.75), whereas five other clusters (HNCL-4, HNCL-5, HNCL-8, HNCL-9, HNCL-11) showed high cluster consensus (consensus ≥ 0.75) but moderate within cluster correlation (0.5 ≤ correlation < 0.75). Cluster HNCL-10 had moderate cluster consensus (consensus = 0.65) and poor cluster correlation (correlation = 0.04), whereas clusters HNCL-3 showed poor cluster consensus (consensus = 0.41) and correlation (correlation = 0.12) (see Table 2).

### Similarity between Lung and H∓N clusters

**Figure 4:**
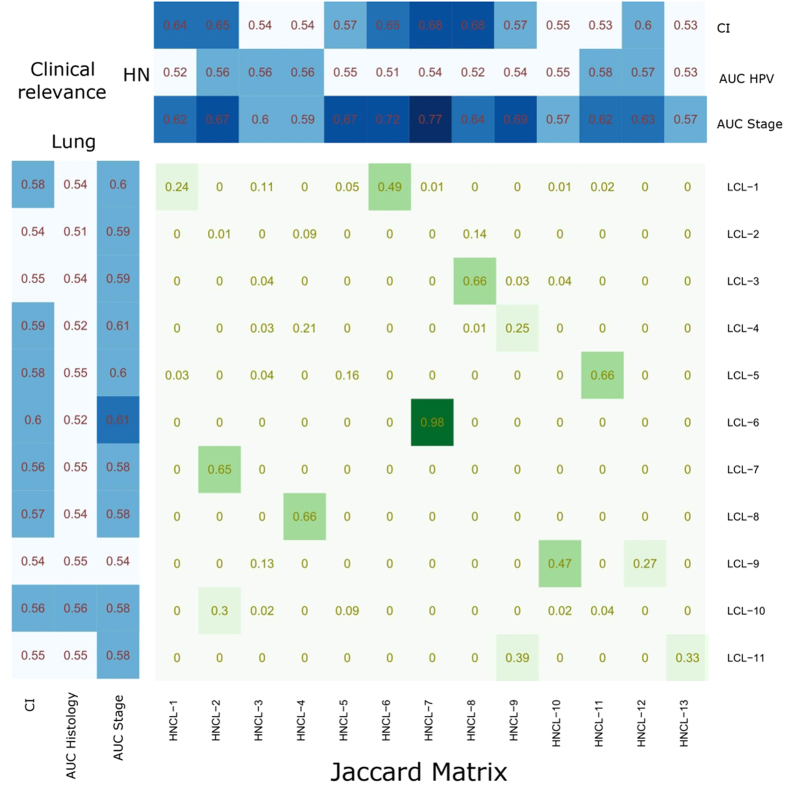
Heatmap depicting cluster overlap and clinical relevance (CI & AUC). Center matrix in green & white color represents the overlap (Jaccard index) between the clusters of Lung (rows) and H & N (columns) radiomic cohorts. Top and left side panels in blue & white color depicts the average CI & AUC of the corresponding Lung and H & N radiomic clusters.

In order to assess the overlap between individual Lung and H & N radiomic clusters, we compared the Lung and H & N clusters, pairwise, using the Jaccard index. We observed that Lung cluster LCL-6 and H & N cluster HNCL-7 had high overlap (Jaccard = 0.98). Cluster pairs LCL-7 & HNCL-2 (Jaccard = 0.65), LCL-8 & HNCL-4 (Jaccard = 0.66) and LCL-5 & HNCL-11 (Jaccard = 0.66) and LCL-3 & HNCL-8 (Jaccard = 0.66) showed moderate overlap, whereas the remaining pairs had poor overlap (Jaccard < 0.5) (see Fig. 4).

### Clinical relevance of radiomic clusters

Mean CI and mean AUC values for the obtained Lung and H & N clusters are depicted in Table 1 & Table 2. All eleven Lung clusters had a significant prognostic association with patient survival. However, only four Lung clusters (LCL-1, LCL-4, LCL-5 and LCL-6) had a mean CI higher or equal to 0.58. Two Lung clusters (LCL-9 and LCL-10) were significantly associated with tumor histology. All Lung clusters had significant association with tumor stage (see Table 1). For H & N cancer, six clusters (HNCL-1, HNCL-2, HNCL-6, HNCL-7, HNCL-8 and HNCL-12) were significantly associated with patient survival. We did not observe any association between the H & N clusters and HPV status. However, except for the three H & N clusters (HNCL-4, HNCL-10, HNCL-13), all the other H & N clusters were significantly associated with tumor stage (see Table 2). Univariable CI and AUC values of radiomic features in Lung1 and HN1 cohorts are represented by heatmaps in Fig. 3e,f, respectively.

### Multivariable clinical relevance

We built two radiomic signatures, one each for Lung and H & N cohort, using the medoids of the obtained Lung and H & N radiomic feature clusters, respectively. To evaluate the multivariate prognostic performance, we trained a multivariable Cox proportional hazards model on both training cohorts (Lung1 and HN1). Prognostic performance of each model was externally tested on validation cohorts (Lung2 and HN2). We observed that the prognostic performance of Lung based multivariable model (CI = 0.61) was higher than the H & N model (CI = 0.56) in Lung2 validation cohort, whereas for HN2 validation cohort, H & N based multivariable model (CI = 0.63) performed better than the Lung model (CI = 0.57). We also used the cluster medoids for the prediction of clinical parameters. We trained classifiers on training cohort (i.e. Lung1 or HN1) using logistic regression and evaluated the predictive performance in the corresponding validation cohort (Lung2 or HN2). The Lung signature had a moderate performance for the prediction of tumor histology (AUC = 0.64) and tumor stage (AUC = 0.64). The H & N signature was highly predictive for tumor stage (AUC = 0.80) and had a moderate predictive performance for HPV status (AUC = 0.60).

## Discussion

Medical imaging plays an important role in medical care and science due to its ability to assess tissue characteristics and organ anatomy non-invasively. It is therefore widely used in disease diagnosis, progression assessment and treatment monitoring in clinical oncology. Radiomics, a high throughput approach, can quantify the differences between oncologic tissues and hence provide prognostic or predictive imaging biomarkers^7^,^8^.

In this study we investigated clustering as a means to deal with the high dimensional feature space generated with radiomics, as well as to investigate common and cancer-type specific radiomic patterns. We applied consensus clustering on 440 radiomic features extracted from Lung cancer and Head & Neck cancer patient cohorts. Furthermore these clusters were externally validated on independent validation cohorts. For both cancer types, many clusters showed high cluster consensus and high within cluster correlation, which indicates the high robustness (stability) and compactness of these clusters. These results indicate that consensus clustering could provide robust radiomic feature clusters and hence reduce the feature redundancy. The majority of the obtained Lung and H∓N radiomic clusters were significantly associated with patient survival and tumor stage. Two Lung clusters also showed significant association with tumor histology. Our multivariable analysis showed that cancer-specific multivariable radiomic signatures displayed moderate or high prognostic (predictive) performance.

Comparing the individual Lung and H & N feature clusters, we observed that five cluster pairs had substantial overlap (Jaccard ≥ 0.6) between the Lung and H∓N cancer, whereas the overlap for other cluster pairs was poor. These results demonstrate both common as well as cancer-specific clustering characteristics of radiomic features.

It can be observed from our analysis that radiomic features also have cancer-specific prognostic ability. We compared the univariable CI values of radiomic features across the two cancer types and observed that several radiomic features have significant prognostic utility in only one of the two cancer types. Furthermore the multivariable radiomic signatures performed better in validation cohorts of the same cancer type in our multivariable analysis.

Recently, Aerts *et al.*^18^, built a radiomic signature of four features, which were selected based on their stability across test-retest image scans and multiple tumor delineations, as well as their prognostic performance in a training dataset. However, the feature selection was only applied to one cancer type (i.e. lung cancer patients). The primary objective of our study was to separately investigate and compare radiomic feature subgroups in Lung and H∓N cancer. Our analysis reveals a cancer-specific grouping and prognostic trends of radiomic features, which could be exploited to potentially improve the performance of prognostic models. In another radiomic study of Lung cancer cohorts, Balagurunathan *et al.*^31^, used a three step procedure to select the most reproducible, informative and non-redundant features. In this method, it was required to empirically choose three threshold values corresponding to the concordance correlation coefficient, dynamic range and pairwise correlation. On the contrary, our method of feature selection only depends on the number of clusters. In general, the estimation of number of clusters is done by a visual inspection of consensus matrices and the delta area plot^20^. However, we used a more deterministic method for the estimation of number of clusters. Overall, the parameters of consensus clustering procedure were carefully chosen, keeping in mind the larger applicability of the obtained clustering results in radiomics. However, these parameters are still just design choices and there may be other relevant parameter configurations, which could be tried and the resultant clustering outputs could be analyzed and compared. These interesting research issues do not fall within the scope of this study, which is primarily focused on the cancer specific comparison of radiomic features.

We expect that the obtained feature clusters of our study combined with feature stability information could further enhance the feature selection process, providing more reliable and prognostic radiomic signatures. However, due to the unavailability of test-retest and multiple delineation H∓N cancer cohorts, we could not compute the stability of H∓N based radiomic features and hence could not include a cancer-specific stability analysis in our study. Machine learning based advanced feature selection methods could also enhance the radiomic biomarker selection process. Although identifying the optimal feature selection method is very important for radiomics research, it does not fit well within the clustering framework of this study and hence should be addressed in future studies, investigating machine learning methods for radiomic data.

We focused only on CT derived radiomic features in this study. However, imaging is routinely performed in clinical practice using different modalities (e.g. CT, PET, MRI, etc.). An integrated radiomic analysis of different cancer types using multiple modalities could therefore provide even more information to facilitate medical decision support with imaging biomarkers.

This study identified different Lung and H & N radiomic feature subgroups and quantified their clinical significance. Our investigation revealed that clustering and prognostic characteristics of radiomic features are cancer-specific. Cancer-specific prognostic characteristics of radiomic features should be considered for building prognostic models, which could improve the prognosis in cancer care. In general, high throughput medical image data mining research like “Radiomics” can influence the cancer research greatly, as it provides a promising non-invasive way of quantifying and monitoring tumor phenotypic characteristics across different cancer types in clinical oncology.

## Additional Information

**How to cite this article**: Parmar, C. *et al.* Radiomic feature clusters and Prognostic Signatures specific for Lung and Head & Neck cancer. *Sci. Rep.* **5**, 11044; doi: 10.1038/srep11044 (2015).

## Supplementary Material

Supplementary Information

## Figures and Tables

**Table 1:**
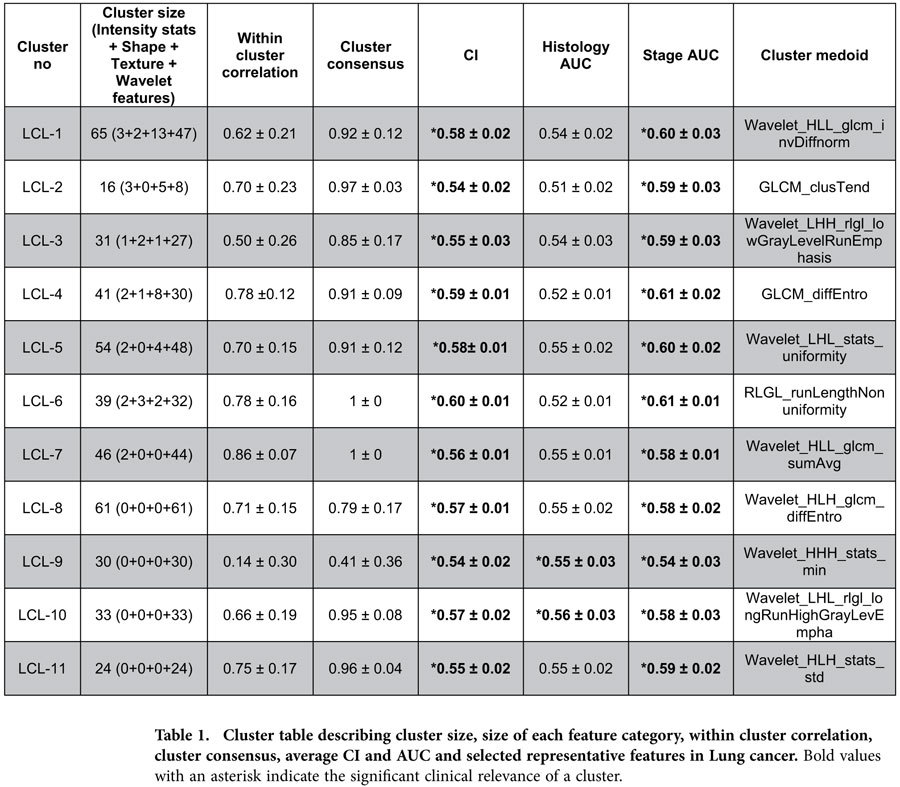
Cluster table describing cluster size, size of each feature category, within cluster correlation, cluster consensus, average CI and AUC and selected representative features in Lung cancer.

**Table 2:**
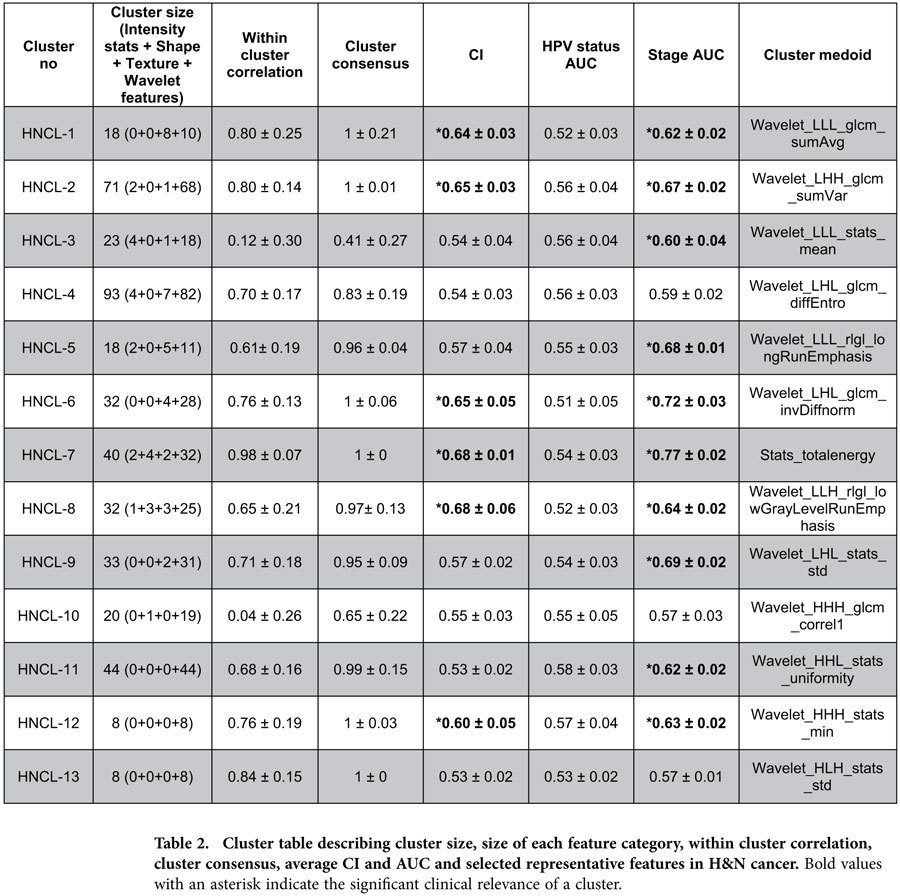
Cluster table describing cluster size, size of each feature category, within cluster correlation, cluster consensus, average CI and AUC and selected representative features in H & N cancer.
