# Supplementary material for: Radiomic feature clusters and Prognostic Signatures specific for Lung and Head & Neck cancer
Source: Sci Rep. 2015 Jun 5;5:11044. doi: 10.1038/srep11044 (PMC4937496; doi:10.1038/srep11044)
Supplement: Supplementary Information [file srep11044-s1.pdf]

Radiomic feature clusters and Prognostic Signatures specific for Lung and Head & Neck cancer

Chintan Parmar[1,3,4,#,*], Ralph T. H. Leijenaar[3,#], Patrick Grossmann[1,5], Emmanuel Rios Velazquez[1], Johan Bussink[6], Derek Rietveld[7], Michelle M. Rietbergen[8], Benjamin Haibe-Kains[9,10], Philippe Lambin[2],

Hugo J.W.L. Aerts[1,2,5,*]

Departments of [1]Radiation Oncology and [2]Radiology, Dana-Farber Cancer Institute, Brigham and Women's Hospital, Harvard Medical School, Boston, MA, USA, [3]Radiation Oncology (MAASTRO), Research Institute GROW, Maastricht University, Maastricht, the Netherlands, [4]Machine Intelligence Unit, Indian Statistical Institute, Kolkata, India, [5]Department of Biostatistics & Computational Biology, Dana-Farber Cancer Institute, Boston, MA, USA, [6]Department of Radiation Oncology, Radboud University Medical Center, Nijmegen, the Netherlands, [7]Department of Radiation Oncology, VU University Medical Center, Amsterdam, The Netherlands, [8]Department of Otolaryngology/Head and Neck Surgery, VU University Medical Center, Amsterdam, The Netherlands, [9]Ontario Cancer Institute, Princess Margaret Cancer Centre, University Health Network, Toronto, Ontario, Canada, [10]Medical Biophysics Department, University of Toronto, Toronto, Ontario, Canada

#Equal contribution

Subject areas:

QUANTITATIVE IMAGING, RADIOLOGY, RADIOMICS, CANCER, COMPUTATIONAL SCIENCE

CORRESPONDING AUTHORS

Hugo Aerts, PhD

Dana-Farber Cancer Institute, Brigham and Women's Hospital, Harvard Medical School

450 Brookline Ave, JF518, Boston, MA 02115-5450, P - 617.525.7156,

F - 617.582.6037

Email: Hugo_Aerts@dfci.harvard.edu

Chintan Parmar, MTech.

Dana-Farber Cancer Institute

450 Brookline Ave, JF518, Boston, MA 02115-5450,

P - 617.525.7156,

F – 617.582.6037

Email: Chintan_Parmar@dfci.harvard.edu

Supplementary Information

Figure S1

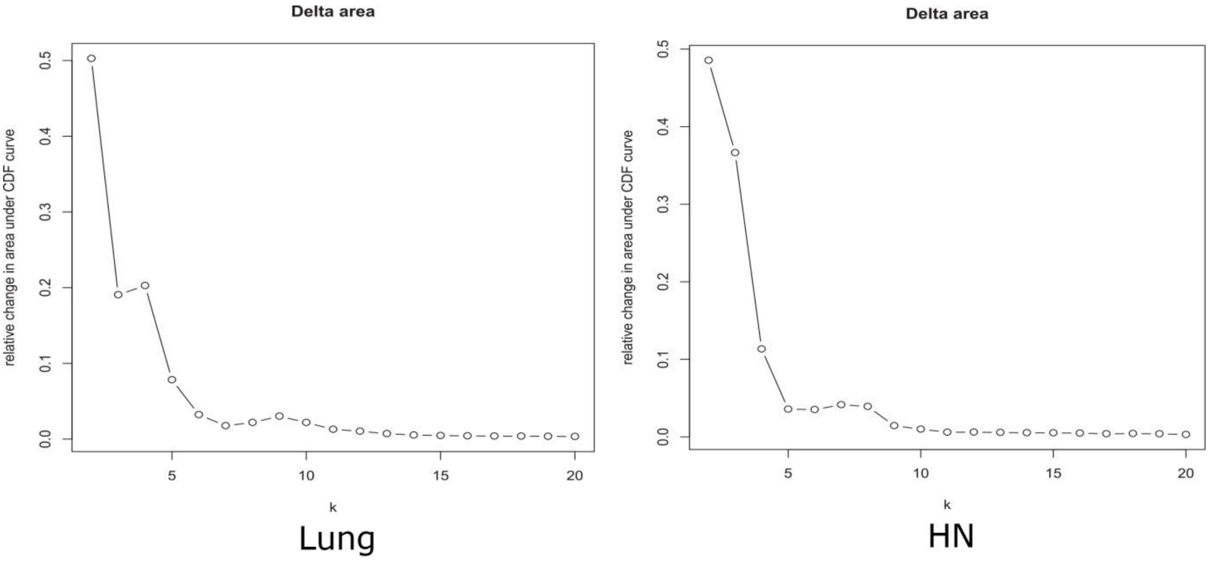

Consensus clustering delta area plots for the Lung1 (left) and HN1 (right) radiomic cohorts. It can be observed that for both Lung1 and HN1 cohorts, the relative change in the area under CDF curves (y-axis) approximately converges to minimum at 10 or more number of clusters (x-axis). Therefore, we chose the number of cluster, which is ≥ 10 and which gave the highest median cluster consensus over all clusters.

Supplementary Table- ST1

Supplementary table listing all the radiomic features along with the corresponding Lung and HN cluster number and CI values.

Serial No.	FeatureName	Lung Cluster Number	Lung CI	Lung CI rank	HN Cluster Number	HN CI	HN CI rank
1	GLCM_autocorr	1	0.57	247	1	0.64	152
2	GLCM_clusProm	2	0.51	434	2	0.61	185
3	GLCM_clusShade	3	0.50	437	3	0.54	331
4	GLCM_clusTend	2	0.54	398	4	0.58	228
5	GLCM_contrast	4	0.58	192	4	0.53	347
6	GLCM_correl1	1	0.56	303	1	0.65	145
7	GLCM_diffEntro	4	0.59	104	4	0.53	356
8	GLCM_dissimilar	4	0.58	128	4	0.51	407
9	GLCM_energy	5	0.58	204	5	0.54	333
10	GLCM_entrop2	4	0.57	242	4	0.53	346
11	GLCM_homogeneity1	1	0.59	124	5	0.57	243
12	GLCM_homogeneity2	1	0.58	131	5	0.57	252
13	GLCM_infoCorr1	1	0.55	367	6	0.53	355
14	GLCM_infoCorr2	4	0.56	289	4	0.52	363
15	GLCM_invDiffmomnor	1	0.59	78	6	0.70	18
16	GLCM_invDiffnorm	1	0.59	59	6	0.70	17
17	GLCM_inverseVar	1	0.59	117	6	0.52	362
18	GLCM_maxProb	5	0.57	254	5	0.55	281
19	GLCM_sumAvg	1	0.57	263	1	0.63	161
20	GLCM_sumEntro	4	0.57	223	4	0.53	344
21	GLCM_sumSquares	1	0.57	258	1	0.64	151
22	GLCM_sumVar	1	0.57	249	1	0.64	154
23	RLGL_grayLevelNonuniformity	6	0.61	8	7	0.67	98
24	RLGL_highGrayLevelRunEmphasis	1	0.57	230	1	0.64	150

25	RLGL_longRunEmphasis	5	0.58	167	5	0.62	178
26	RLGL_longRunHighGrayLevEmpha	5	0.58	175	1	0.66	120
27	RLGL_longRunLowGrayLevEmpha	2	0.53	407	8	0.54	309
28	RLGL_lowGrayLevelRunEmphasis	2	0.55	359	8	0.66	127
29	RLGL_runLengthNonuniformity	6	0.60	37	7	0.68	39
30	RLGL_runPercentage	4	0.58	135	9	0.62	175
31	RLGL_shortRunEmphasis	4	0.59	113	9	0.61	191
32	RLGL_shortRunHighGrayLevEmpha	1	0.56	338	1	0.57	242
33	RLGL_shortRunLowGrayLevEmpha	2	0.56	332	8	0.67	94
34	Shape_compactness	6	0.60	25	7	0.68	75
35	Shape_compactness2	3	0.55	354	8	0.58	218
36	Shape_maxDiameter	6	0.60	43	7	0.68	54
37	Shape_spherDisprop	1	0.55	356	10	0.59	216
38	Shape_sphericity	3	0.55	355	8	0.58	223
39	Shape_surfVolRatio	4	0.60	52	8	0.67	104
40	Shape_surface	6	0.60	31	7	0.68	32
41	Shape_volume	1	0.53	406	7	0.68	53
42	Stats_energy	6	0.61	15	7	0.68	65
43	Stats_entropy	4	0.58	180	4	0.50	440
44	Stats_kurtosis	5	0.58	190	5	0.58	225
45	Stats_max	7	0.57	225	2	0.66	124
46	Stats_md	2	0.56	311	4	0.52	368
47	Stats_mean	1	0.58	139	3	0.51	421
48	Stats_median	1	0.61	5	3	0.50	424
49	Stats_min	3	0.54	397	8	0.66	111
50	Stats_range	7	0.57	245	2	0.66	117
51	Stats_rms	1	0.59	85	3	0.53	337
52	Stats_skewness	4	0.56	293	3	0.52	382
53	Stats_std	2	0.54	380	4	0.57	258
54	Stats_totalenergy	6	0.61	17	7	0.68	61
55	Stats_uniformity	5	0.58	147	5	0.54	320

56	Stats_var	2	0.54	379	4	0.57	257
57	Wavelet_HHH_glcm_autocorr	7	0.56	305	2	0.68	44
58	Wavelet_HHH_glcm_clusProm	8	0.54	383	4	0.61	194
59	Wavelet_HHH_glcm_clusShade	9	0.51	431	10	0.52	367
60	Wavelet_HHH_glcm_clusTend	8	0.56	328	4	0.54	307
61	Wavelet_HHH_glcm_contrast	8	0.56	334	4	0.55	297
62	Wavelet_HHH_glcm_correl1	9	0.54	389	10	0.54	311
63	Wavelet_HHH_glcm_diffEntro	8	0.56	339	4	0.54	306
64	Wavelet_HHH_glcm_dissimilar	8	0.56	325	4	0.54	332
65	Wavelet_HHH_glcm_energy	10	0.54	386	11	0.56	270
66	Wavelet_HHH_glcm_entrop2	8	0.53	405	4	0.59	210
67	Wavelet_HHH_glcm_homogeneity1	5	0.56	326	11	0.53	354
68	Wavelet_HHH_glcm_homogeneity2	5	0.56	330	11	0.53	357
69	Wavelet_HHH_glcm_infoCorr1	1	0.59	75	6	0.63	167
70	Wavelet_HHH_glcm_infoCorr2	4	0.60	42	9	0.58	229
71	Wavelet_HHH_glcm_invDiffmomnor	1	0.59	95	6	0.72	12
72	Wavelet_HHH_glcm_invDiffnorm	1	0.59	122	6	0.67	91
73	Wavelet_HHH_glcm_inverseVar	5	0.56	336	11	0.51	393
74	Wavelet_HHH_glcm_maxProb	10	0.53	411	11	0.56	275
75	Wavelet_HHH_glcm_sumAvg	7	0.56	307	2	0.68	46
76	Wavelet_HHH_glcm_sumEntro	8	0.56	340	4	0.55	300
77	Wavelet_HHH_glcm_sumSquares	7	0.56	318	2	0.68	56
78	Wavelet_HHH_glcm_sumVar	7	0.56	301	2	0.68	45
79	Wavelet_HHH_rlgl_grayLevelNonuniformity	6	0.61	7	7	0.67	100
80	Wavelet_HHH_rlgl_highGrayLevelRunEmphasis	7	0.56	315	2	0.68	57
81	Wavelet_HHH_rlgl_longRunEmphasis	10	0.57	237	11	0.52	387
82	Wavelet_HHH_rlgl_longRunHighGrayLevEmpha	10	0.57	262	2	0.68	55
83	Wavelet_HHH_rlgl_longRunLowGrayLevEmpha	3	0.56	319	8	0.70	19
84	Wavelet_HHH_rlgl_lowGrayLevelRunEmphasis	3	0.57	220	8	0.73	8
85	Wavelet_HHH_rlgl_runLengthNonuniformity	6	0.60	33	7	0.68	40
86	Wavelet_HHH_rlgl_runPercentage	8	0.57	234	4	0.51	396

87	Wavelet_HHH_rlgl_shortRunEmphasis	8	0.57	232	4	0.51	394
88	Wavelet_HHH_rlgl_shortRunHighGrayLevEmpha	7	0.56	342	2	0.68	63
89	Wavelet_HHH_rlgl_shortRunLowGrayLevEmpha	3	0.58	207	8	0.77	3
90	Wavelet_HHH_stats_energy	6	0.60	53	7	0.67	102
91	Wavelet_HHH_stats_entropy	8	0.56	310	4	0.54	330
92	Wavelet_HHH_stats_kurtosis	10	0.55	369	2	0.64	149
93	Wavelet_HHH_stats_max	10	0.57	266	2	0.58	226
94	Wavelet_HHH_stats_md	11	0.52	422	9	0.59	215
95	Wavelet_HHH_stats_mean	3	0.52	421	10	0.52	380
96	Wavelet_HHH_stats_median	3	0.50	440	10	0.51	417
97	Wavelet_HHH_stats_min	9	0.56	287	12	0.60	199
98	Wavelet_HHH_stats_range	10	0.57	253	2	0.60	203
99	Wavelet_HHH_stats_rms	11	0.51	427	9	0.57	245
100	Wavelet_HHH_stats_skewness	9	0.53	413	10	0.54	326
101	Wavelet_HHH_stats_std	11	0.51	429	9	0.57	246
102	Wavelet_HHH_stats_totalenergy	6	0.60	58	7	0.67	101
103	Wavelet_HHH_stats_uniformity	5	0.56	317	11	0.53	351
104	Wavelet_HHH_stats_var	11	0.51	428	9	0.57	248
105	Wavelet_HHL_glcm_autocorr	7	0.56	308	2	0.68	67
106	Wavelet_HHL_glcm_clusProm	8	0.56	300	4	0.57	238
107	Wavelet_HHL_glcm_clusShade	9	0.54	394	10	0.55	286
108	Wavelet_HHL_glcm_clusTend	8	0.58	134	4	0.52	391
109	Wavelet_HHL_glcm_contrast	8	0.58	158	4	0.52	376
110	Wavelet_HHL_glcm_correl1	9	0.55	352	10	0.55	293
111	Wavelet_HHL_glcm_diffEntro	8	0.59	107	4	0.50	437
112	Wavelet_HHL_glcm_dissimilar	8	0.59	120	4	0.50	433
113	Wavelet_HHL_glcm_energy	5	0.58	149	11	0.50	430
114	Wavelet_HHL_glcm_entrop2	8	0.58	173	4	0.54	327
115	Wavelet_HHL_glcm_homogeneity1	5	0.58	137	11	0.51	404
116	Wavelet_HHL_glcm_homogeneity2	5	0.58	148	11	0.51	402
117	Wavelet_HHL_glcm_infoCorr1	1	0.58	143	6	0.61	195

118	Wavelet_HHL_glcm_infoCorr2	4	0.60	45	9	0.57	253
119	Wavelet_HHL_glcm_invDiffmomnor	1	0.61	2	6	0.68	73
120	Wavelet_HHL_glcm_invDiffnorm	1	0.60	21	6	0.66	110
121	Wavelet_HHL_glcm_inverseVar	5	0.59	123	3	0.57	244
122	Wavelet_HHL_glcm_maxProb	5	0.57	224	11	0.51	413
123	Wavelet_HHL_glcm_sumAvg	7	0.56	306	2	0.68	62
124	Wavelet_HHL_glcm_sumEntro	8	0.59	90	4	0.50	438
125	Wavelet_HHL_glcm_sumSquares	7	0.56	329	2	0.68	59
126	Wavelet_HHL_glcm_sumVar	7	0.56	297	2	0.68	64
127	Wavelet_HHL_rlgl_grayLevelNonuniformity	6	0.61	3	7	0.67	92
128	Wavelet_HHL_rlgl_highGrayLevelRunEmphasis	7	0.56	337	2	0.68	58
129	Wavelet_HHL_rlgl_longRunEmphasis	5	0.58	161	11	0.54	323
130	Wavelet_HHL_rlgl_longRunHighGrayLevEmpha	10	0.58	157	2	0.69	27
131	Wavelet_HHL_rlgl_longRunLowGrayLevEmpha	3	0.55	368	8	0.68	60
132	Wavelet_HHL_rlgl_lowGrayLevelRunEmphasis	3	0.57	226	8	0.70	16
133	Wavelet_HHL_rlgl_runLengthNonuniformity	6	0.60	46	7	0.68	29
134	Wavelet_HHL_rlgl_runPercentage	8	0.58	130	4	0.54	319
135	Wavelet_HHL_rlgl_shortRunEmphasis	8	0.59	105	4	0.54	315
136	Wavelet_HHL_rlgl_shortRunHighGrayLevEmpha	7	0.55	370	2	0.67	85
137	Wavelet_HHL_rlgl_shortRunLowGrayLevEmpha	3	0.58	191	8	0.71	13
138	Wavelet_HHL_stats_energy	6	0.59	70	7	0.67	97
139	Wavelet_HHL_stats_entropy	8	0.59	82	4	0.51	412
140	Wavelet_HHL_stats_kurtosis	10	0.60	55	2	0.63	171
141	Wavelet_HHL_stats_max	10	0.57	244	2	0.63	168
142	Wavelet_HHL_stats_md	11	0.57	229	9	0.59	211
143	Wavelet_HHL_stats_mean	9	0.53	414	3	0.52	390
144	Wavelet_HHL_stats_median	9	0.54	381	3	0.67	89
145	Wavelet_HHL_stats_min	9	0.57	221	12	0.58	230
146	Wavelet_HHL_stats_range	10	0.58	188	2	0.61	182
147	Wavelet_HHL_stats_rms	11	0.56	283	9	0.57	237
148	Wavelet_HHL_stats_skewness	10	0.52	426	10	0.56	266

149	Wavelet_HHL_stats_std	11	0.56	286	9	0.58	235
150	Wavelet_HHL_stats_totalenergy	6	0.59	81	7	0.67	95
151	Wavelet_HHL_stats_uniformity	5	0.59	89	11	0.52	378
152	Wavelet_HHL_stats_var	11	0.56	284	9	0.58	236
153	Wavelet_HLH_glcm_autocorr	7	0.56	316	2	0.65	133
154	Wavelet_HLH_glcm_clusProm	8	0.56	323	4	0.63	158
155	Wavelet_HLH_glcm_clusShade	9	0.52	423	3	0.54	334
156	Wavelet_HLH_glcm_clusTend	8	0.58	199	4	0.58	221
157	Wavelet_HLH_glcm_contrast	8	0.57	228	4	0.58	220
158	Wavelet_HLH_glcm_correl1	9	0.54	382	10	0.52	389
159	Wavelet_HLH_glcm_diffEntro	8	0.58	196	4	0.53	336
160	Wavelet_HLH_glcm_dissimilar	8	0.58	189	4	0.54	328
161	Wavelet_HLH_glcm_energy	5	0.58	201	11	0.50	427
162	Wavelet_HLH_glcm_entrop2	8	0.57	259	4	0.55	301
163	Wavelet_HLH_glcm_homogeneity1	5	0.58	184	11	0.50	431
164	Wavelet_HLH_glcm_homogeneity2	5	0.58	186	11	0.50	425
165	Wavelet_HLH_glcm_infoCorr1	1	0.58	160	6	0.61	192
166	Wavelet_HLH_glcm_infoCorr2	4	0.59	71	9	0.55	285
167	Wavelet_HLH_glcm_invDiffmomnor	1	0.60	19	6	0.66	112
168	Wavelet_HLH_glcm_invDiffnorm	1	0.60	27	6	0.66	123
169	Wavelet_HLH_glcm_inverseVar	5	0.58	185	3	0.54	314
170	Wavelet_HLH_glcm_maxProb	5	0.57	246	11	0.52	384
171	Wavelet_HLH_glcm_sumAvg	7	0.56	313	2	0.65	131
172	Wavelet_HLH_glcm_sumEntro	8	0.58	162	4	0.53	339
173	Wavelet_HLH_glcm_sumSquares	7	0.56	327	2	0.65	134
174	Wavelet_HLH_glcm_sumVar	7	0.56	309	2	0.65	137
175	Wavelet_HLH_rlgl_grayLevelNonuniformity	6	0.61	1	7	0.67	84
176	Wavelet_HLH_rlgl_highGrayLevelRunEmphasis	7	0.56	335	2	0.65	132
177	Wavelet_HLH_rlgl_longRunEmphasis	5	0.59	119	11	0.55	294
178	Wavelet_HLH_rlgl_longRunHighGrayLevEmpha	10	0.57	231	2	0.65	146
179	Wavelet_HLH_rlgl_longRunLowGrayLevEmpha	3	0.55	358	8	0.66	115

180	Wavelet_HLH_rlgl_lowGrayLevelRunEmphasis	3	0.57	216	8	0.71	14
181	Wavelet_HLH_rlgl_runLengthNonuniformity	6	0.60	41	7	0.69	20
182	Wavelet_HLH_rlgl_runPercentage	8	0.59	103	4	0.54	304
183	Wavelet_HLH_rlgl_shortRunEmphasis	8	0.59	114	4	0.53	345
184	Wavelet_HLH_rlgl_shortRunHighGrayLevEmpha	7	0.55	361	2	0.65	142
185	Wavelet_HLH_rlgl_shortRunLowGrayLevEmpha	3	0.58	197	8	0.73	9
186	Wavelet_HLH_stats_energy	6	0.59	110	7	0.68	50
187	Wavelet_HLH_stats_entropy	8	0.58	151	4	0.51	400
188	Wavelet_HLH_stats_kurtosis	10	0.58	132	5	0.61	196
189	Wavelet_HLH_stats_max	10	0.56	281	2	0.63	170
190	Wavelet_HLH_stats_md	11	0.57	227	13	0.56	273
191	Wavelet_HLH_stats_mean	9	0.52	425	3	0.51	401
192	Wavelet_HLH_stats_median	9	0.53	409	3	0.55	295
193	Wavelet_HLH_stats_min	9	0.55	347	12	0.59	212
194	Wavelet_HLH_stats_range	10	0.56	290	2	0.61	184
195	Wavelet_HLH_stats_rms	11	0.56	278	13	0.52	370
196	Wavelet_HLH_stats_skewness	10	0.51	432	3	0.55	292
197	Wavelet_HLH_stats_std	11	0.56	276	13	0.52	374
198	Wavelet_HLH_stats_totalenergy	6	0.58	127	7	0.68	47
199	Wavelet_HLH_stats_uniformity	5	0.58	140	11	0.51	405
200	Wavelet_HLH_stats_var	11	0.56	277	13	0.52	372
201	Wavelet_HLL_glcm_autocorr	7	0.55	372	2	0.63	162
202	Wavelet_HLL_glcm_clusProm	8	0.56	331	4	0.60	202
203	Wavelet_HLL_glcm_clusShade	9	0.52	424	10	0.61	183
204	Wavelet_HLL_glcm_clusTend	4	0.58	165	4	0.53	350
205	Wavelet_HLL_glcm_contrast	4	0.58	159	4	0.53	340
206	Wavelet_HLL_glcm_correl1	9	0.54	399	10	0.57	255
207	Wavelet_HLL_glcm_diffEntro	4	0.59	69	4	0.52	379
208	Wavelet_HLL_glcm_dissimilar	4	0.59	80	4	0.51	392
209	Wavelet_HLL_glcm_energy	5	0.60	48	11	0.55	299
210	Wavelet_HLL_glcm_entrop2	4	0.59	108	4	0.50	434

211	Wavelet_HLL_glcm_homogeneity1	1	0.60	49	11	0.55	288
212	Wavelet_HLL_glcm_homogeneity2	1	0.60	50	11	0.55	280
213	Wavelet_HLL_glcm_infoCorr1	1	0.54	384	6	0.60	198
214	Wavelet_HLL_glcm_infoCorr2	4	0.58	153	9	0.57	240
215	Wavelet_HLL_glcm_invDiffmomnor	1	0.60	20	6	0.68	30
216	Wavelet_HLL_glcm_invDiffnorm	1	0.61	12	6	0.65	140
217	Wavelet_HLL_glcm_inverseVar	1	0.59	61	3	0.58	219
218	Wavelet_HLL_glcm_maxProb	5	0.59	65	11	0.54	313
219	Wavelet_HLL_glcm_sumAvg	7	0.55	366	2	0.63	160
220	Wavelet_HLL_glcm_sumEntro	4	0.59	72	4	0.52	377
221	Wavelet_HLL_glcm_sumSquares	7	0.53	401	2	0.63	165
222	Wavelet_HLL_glcm_sumVar	7	0.55	365	2	0.63	157
223	Wavelet_HLL_rlgl_grayLevelNonuniformity	6	0.61	4	7	0.67	76
224	Wavelet_HLL_rlgl_highGrayLevelRunEmphasis	7	0.54	391	2	0.63	159
225	Wavelet_HLL_rlgl_longRunEmphasis	5	0.60	38	11	0.57	241
226	Wavelet_HLL_rlgl_longRunHighGrayLevEmpha	10	0.59	79	2	0.65	138
227	Wavelet_HLL_rlgl_longRunLowGrayLevEmpha	3	0.52	420	8	0.61	190
228	Wavelet_HLL_rlgl_lowGrayLevelRunEmphasis	3	0.58	176	8	0.66	113
229	Wavelet_HLL_rlgl_runLengthNonuniformity	6	0.60	51	7	0.68	51
230	Wavelet_HLL_rlgl_runPercentage	4	0.60	34	4	0.58	232
231	Wavelet_HLL_rlgl_shortRunEmphasis	4	0.60	30	4	0.57	239
232	Wavelet_HLL_rlgl_shortRunHighGrayLevEmpha	7	0.53	412	2	0.62	172
233	Wavelet_HLL_rlgl_shortRunLowGrayLevEmpha	3	0.58	129	8	0.68	72
234	Wavelet_HLL_stats_energy	6	0.59	99	7	0.68	74
235	Wavelet_HLL_stats_entropy	4	0.60	54	4	0.52	365
236	Wavelet_HLL_stats_kurtosis	10	0.60	35	5	0.63	169
237	Wavelet_HLL_stats_max	10	0.58	206	2	0.68	70
238	Wavelet_HLL_stats_md	4	0.59	86	9	0.58	227
239	Wavelet_HLL_stats_mean	1	0.59	91	6	0.69	21
240	Wavelet_HLL_stats_median	1	0.59	97	6	0.63	163
241	Wavelet_HLL_stats_min	9	0.55	363	12	0.62	180

242	Wavelet_HLL_stats_range	10	0.58	194	2	0.66	109
243	Wavelet_HLL_stats_rms	4	0.58	144	9	0.54	303
244	Wavelet_HLL_stats_skewness	9	0.54	396	10	0.63	164
245	Wavelet_HLL_stats_std	4	0.58	154	9	0.54	317
246	Wavelet_HLL_stats_totalenergy	6	0.59	106	7	0.68	71
247	Wavelet_HLL_stats_uniformity	5	0.60	39	11	0.55	298
248	Wavelet_HLL_stats_var	4	0.58	155	9	0.54	318
249	Wavelet_LHH_glcm_autocorr	7	0.56	282	2	0.67	81
250	Wavelet_LHH_glcm_clusProm	8	0.57	265	4	0.61	193
251	Wavelet_LHH_glcm_clusShade	9	0.50	439	3	0.51	410
252	Wavelet_LHH_glcm_clusTend	8	0.57	213	4	0.55	290
253	Wavelet_LHH_glcm_contrast	8	0.56	269	4	0.55	279
254	Wavelet_LHH_glcm_correl1	9	0.56	270	10	0.51	408
255	Wavelet_LHH_glcm_diffEntro	8	0.56	274	4	0.55	287
256	Wavelet_LHH_glcm_dissimilar	8	0.56	288	4	0.54	305
257	Wavelet_LHH_glcm_energy	5	0.55	374	11	0.57	247
258	Wavelet_LHH_glcm_entrop2	8	0.55	376	4	0.58	222
259	Wavelet_LHH_glcm_homogeneity1	5	0.56	341	11	0.55	282
260	Wavelet_LHH_glcm_homogeneity2	5	0.55	344	11	0.55	284
261	Wavelet_LHH_glcm_infoCorr1	1	0.59	88	6	0.62	177
262	Wavelet_LHH_glcm_infoCorr2	4	0.60	47	9	0.56	261
263	Wavelet_LHH_glcm_invDiffmomnor	1	0.59	115	6	0.72	11
264	Wavelet_LHH_glcm_invDiffnorm	1	0.58	156	6	0.69	23
265	Wavelet_LHH_glcm_inverseVar	5	0.56	333	11	0.55	296
266	Wavelet_LHH_glcm_maxProb	5	0.53	404	11	0.58	234
267	Wavelet_LHH_glcm_sumAvg	7	0.56	280	2	0.67	80
268	Wavelet_LHH_glcm_sumEntro	8	0.57	240	4	0.55	289
269	Wavelet_LHH_glcm_sumSquares	7	0.56	294	2	0.67	83
270	Wavelet_LHH_glcm_sumVar	7	0.56	273	2	0.67	79
271	Wavelet_LHH_rlgl_grayLevelNonuniformity	6	0.61	10	7	0.67	107
272	Wavelet_LHH_rlgl_highGrayLevelRunEmphasis	7	0.56	291	2	0.67	82

273	Wavelet_LHH_rlgl_longRunEmphasis	5	0.57	256	11	0.51	414
274	Wavelet_LHH_rlgl_longRunHighGrayLevEmpha	10	0.57	239	2	0.69	22
275	Wavelet_LHH_rlgl_longRunLowGrayLevEmpha	3	0.56	302	8	0.68	33
276	Wavelet_LHH_rlgl_lowGrayLevelRunEmphasis	3	0.57	219	8	0.67	90
277	Wavelet_LHH_rlgl_runLengthNonuniformity	6	0.60	32	7	0.68	37
278	Wavelet_LHH_rlgl_runPercentage	8	0.57	251	4	0.51	422
279	Wavelet_LHH_rlgl_shortRunEmphasis	8	0.57	248	4	0.51	403
280	Wavelet_LHH_rlgl_shortRunHighGrayLevEmpha	7	0.56	314	2	0.67	88
281	Wavelet_LHH_rlgl_shortRunLowGrayLevEmpha	3	0.58	203	8	0.67	105
282	Wavelet_LHH_stats_energy	6	0.59	83	7	0.68	49
283	Wavelet_LHH_stats_entropy	8	0.57	241	4	0.54	322
284	Wavelet_LHH_stats_kurtosis	10	0.55	346	2	0.62	174
285	Wavelet_LHH_stats_max	10	0.56	322	2	0.60	207
286	Wavelet_LHH_stats_md	11	0.53	410	9	0.58	224
287	Wavelet_LHH_stats_mean	3	0.55	364	9	0.60	201
288	Wavelet_LHH_stats_median	3	0.50	435	9	0.54	329
289	Wavelet_LHH_stats_min	9	0.56	285	12	0.61	188
290	Wavelet_LHH_stats_range	10	0.56	279	2	0.61	186
291	Wavelet_LHH_stats_rms	11	0.53	418	9	0.57	260
292	Wavelet_LHH_stats_skewness	9	0.53	400	12	0.51	419
293	Wavelet_LHH_stats_std	11	0.53	417	9	0.56	263
294	Wavelet_LHH_stats_totalenergy	6	0.59	87	7	0.68	38
295	Wavelet_LHH_stats_uniformity	5	0.56	268	11	0.54	310
296	Wavelet_LHH_stats_var	11	0.53	416	9	0.56	262
297	Wavelet_LHL_glcm_autocorr	7	0.55	362	2	0.65	128
298	Wavelet_LHL_glcm_clusProm	8	0.56	320	4	0.59	213
299	Wavelet_LHL_glcm_clusShade	9	0.52	419	10	0.52	364
300	Wavelet_LHL_glcm_clusTend	8	0.58	126	4	0.53	360
301	Wavelet_LHL_glcm_contrast	8	0.58	183	4	0.53	341
302	Wavelet_LHL_glcm_correl1	9	0.55	353	10	0.53	338
303	Wavelet_LHL_glcm_diffEntro	8	0.58	136	4	0.50	428

304	Wavelet_LHL_glcm_dissimilar	8	0.58	145	4	0.50	439
305	Wavelet_LHL_glcm_energy	5	0.58	166	11	0.51	415
306	Wavelet_LHL_glcm_entrop2	8	0.58	169	4	0.53	343
307	Wavelet_LHL_glcm_homogeneity1	5	0.58	171	11	0.51	409
308	Wavelet_LHL_glcm_homogeneity2	5	0.58	178	11	0.51	418
309	Wavelet_LHL_glcm_infoCorr1	1	0.58	193	6	0.61	189
310	Wavelet_LHL_glcm_infoCorr2	4	0.59	94	9	0.56	278
311	Wavelet_LHL_glcm_invDiffmomnor	1	0.59	67	6	0.73	7
312	Wavelet_LHL_glcm_invDiffnorm	1	0.59	74	6	0.66	116
313	Wavelet_LHL_glcm_inverseVar	1	0.58	138	3	0.56	265
314	Wavelet_LHL_glcm_maxProb	5	0.58	202	11	0.51	420
315	Wavelet_LHL_glcm_sumAvg	7	0.55	357	2	0.65	129
316	Wavelet_LHL_glcm_sumEntro	8	0.59	98	4	0.50	435
317	Wavelet_LHL_glcm_sumSquares	7	0.55	371	2	0.65	136
318	Wavelet_LHL_glcm_sumVar	7	0.55	351	2	0.65	130
319	Wavelet_LHL_rlgl_grayLevelNonuniformity	6	0.61	11	7	0.67	87
320	Wavelet_LHL_rlgl_highGrayLevelRunEmphasis	7	0.55	375	2	0.65	135
321	Wavelet_LHL_rlgl_longRunEmphasis	5	0.58	182	11	0.54	316
322	Wavelet_LHL_rlgl_longRunHighGrayLevEmpha	10	0.57	217	2	0.65	139
323	Wavelet_LHL_rlgl_longRunLowGrayLevEmpha	3	0.54	385	8	0.66	119
324	Wavelet_LHL_rlgl_lowGrayLevelRunEmphasis	3	0.57	255	8	0.73	10
325	Wavelet_LHL_rlgl_runLengthNonuniformity	6	0.60	36	7	0.69	25
326	Wavelet_LHL_rlgl_runPercentage	8	0.58	163	4	0.54	321
327	Wavelet_LHL_rlgl_shortRunEmphasis	8	0.58	146	4	0.53	342
328	Wavelet_LHL_rlgl_shortRunHighGrayLevEmpha	7	0.54	388	2	0.65	143
329	Wavelet_LHL_rlgl_shortRunLowGrayLevEmpha	3	0.57	238	8	0.75	5
330	Wavelet_LHL_stats_energy	6	0.59	121	7	0.68	48
331	Wavelet_LHL_stats_entropy	8	0.59	93	4	0.50	432
332	Wavelet_LHL_stats_kurtosis	10	0.59	73	5	0.61	181
333	Wavelet_LHL_stats_max	10	0.55	360	2	0.61	187
334	Wavelet_LHL_stats_md	11	0.58	181	9	0.59	214

335	Wavelet_LHL_stats_mean	1	0.61	14	6	0.67	96
336	Wavelet_LHL_stats_median	1	0.60	29	6	0.67	108
337	Wavelet_LHL_stats_min	9	0.55	377	12	0.59	209
338	Wavelet_LHL_stats_range	10	0.56	343	2	0.62	179
339	Wavelet_LHL_stats_rms	11	0.57	250	9	0.56	264
340	Wavelet_LHL_stats_skewness	9	0.53	415	10	0.50	436
341	Wavelet_LHL_stats_std	11	0.57	260	9	0.56	269
342	Wavelet_LHL_stats_totalenergy	6	0.58	142	7	0.68	42
343	Wavelet_LHL_stats_uniformity	5	0.59	100	11	0.51	398
344	Wavelet_LHL_stats_var	11	0.57	261	9	0.56	268
345	Wavelet_LLH_glcm_autocorr	7	0.56	275	2	0.68	36
346	Wavelet_LLH_glcm_clusProm	8	0.55	350	4	0.64	153
347	Wavelet_LLH_glcm_clusShade	9	0.53	403	10	0.56	274
348	Wavelet_LLH_glcm_clusTend	8	0.58	198	4	0.58	233
349	Wavelet_LLH_glcm_contrast	8	0.57	243	4	0.57	254
350	Wavelet_LLH_glcm_correl1	9	0.55	378	10	0.54	302
351	Wavelet_LLH_glcm_diffEntro	8	0.58	205	4	0.51	406
352	Wavelet_LLH_glcm_dissimilar	8	0.58	208	4	0.51	395
353	Wavelet_LLH_glcm_energy	5	0.58	200	11	0.53	358
354	Wavelet_LLH_glcm_entrop2	8	0.57	235	4	0.52	385
355	Wavelet_LLH_glcm_homogeneity1	5	0.57	214	11	0.52	381
356	Wavelet_LLH_glcm_homogeneity2	5	0.57	218	11	0.52	373
357	Wavelet_LLH_glcm_infoCorr1	1	0.57	215	6	0.58	231
358	Wavelet_LLH_glcm_infoCorr2	4	0.59	101	9	0.54	308
359	Wavelet_LLH_glcm_invDiffmomnor	1	0.59	64	6	0.64	147
360	Wavelet_LLH_glcm_invDiffnorm	1	0.59	68	6	0.63	156
361	Wavelet_LLH_glcm_inverseVar	5	0.58	172	3	0.53	361
362	Wavelet_LLH_glcm_maxProb	5	0.57	252	11	0.54	324
363	Wavelet_LLH_glcm_sumAvg	7	0.56	272	2	0.68	35
364	Wavelet_LLH_glcm_sumEntro	8	0.59	111	4	0.50	429
365	Wavelet_LLH_glcm_sumSquares	7	0.56	295	2	0.68	41

366	Wavelet_LLH_glcm_sumVar	7	0.56	267	2	0.68	31
367	Wavelet_LLH_rlgl_grayLevelNonuniformity	6	0.61	6	7	0.67	86
368	Wavelet_LLH_rlgl_highGrayLevelRunEmphasis	7	0.56	296	2	0.68	52
369	Wavelet_LLH_rlgl_longRunEmphasis	5	0.58	195	11	0.57	249
370	Wavelet_LLH_rlgl_longRunHighGrayLevEmpha	10	0.58	152	2	0.69	26
371	Wavelet_LLH_rlgl_longRunLowGrayLevEmpha	3	0.55	348	8	0.67	78
372	Wavelet_LLH_rlgl_lowGrayLevelRunEmphasis	3	0.57	211	8	0.78	1
373	Wavelet_LLH_rlgl_runLengthNonuniformity	6	0.60	44	7	0.69	24
374	Wavelet_LLH_rlgl_runPercentage	8	0.58	179	4	0.57	259
375	Wavelet_LLH_rlgl_shortRunEmphasis	8	0.58	168	4	0.55	283
376	Wavelet_LLH_rlgl_shortRunHighGrayLevEmpha	7	0.55	345	2	0.67	77
377	Wavelet_LLH_rlgl_shortRunLowGrayLevEmpha	3	0.58	187	8	0.77	2
378	Wavelet_LLH_stats_energy	6	0.58	164	7	0.68	34
379	Wavelet_LLH_stats_entropy	8	0.59	116	4	0.51	411
380	Wavelet_LLH_stats_kurtosis	10	0.59	92	5	0.62	176
381	Wavelet_LLH_stats_max	10	0.54	395	2	0.59	208
382	Wavelet_LLH_stats_md	11	0.59	109	13	0.54	312
383	Wavelet_LLH_stats_mean	1	0.58	141	1	0.57	251
384	Wavelet_LLH_stats_median	1	0.59	63	1	0.60	205
385	Wavelet_LLH_stats_min	9	0.55	349	12	0.68	43
386	Wavelet_LLH_stats_range	10	0.55	373	2	0.65	144
387	Wavelet_LLH_stats_rms	11	0.57	212	13	0.51	416
388	Wavelet_LLH_stats_skewness	9	0.56	271	10	0.57	250
389	Wavelet_LLH_stats_std	11	0.57	209	13	0.51	397
390	Wavelet_LLH_stats_totalenergy	6	0.58	174	7	0.68	28
391	Wavelet_LLH_stats_uniformity	5	0.59	125	11	0.53	349
392	Wavelet_LLH_stats_var	11	0.57	210	13	0.51	399
393	Wavelet_LLL_glcm_autocorr	1	0.56	304	1	0.66	125
394	Wavelet_LLL_glcm_clusProm	2	0.50	436	4	0.62	173
395	Wavelet_LLL_glcm_clusShade	3	0.50	438	3	0.55	291
396	Wavelet_LLL_glcm_clusTend	2	0.54	387	4	0.60	206

397	Wavelet_LLL_glcm_contrast	4	0.59	112	4	0.53	348
398	Wavelet_LLL_glcm_correl1	1	0.57	257	1	0.64	155
399	Wavelet_LLL_glcm_diffEntro	4	0.60	57	4	0.52	366
400	Wavelet_LLL_glcm_dissimilar	4	0.59	60	4	0.52	386
401	Wavelet_LLL_glcm_energy	5	0.59	76	5	0.52	371
402	Wavelet_LLL_glcm_entrop2	4	0.58	133	4	0.52	369
403	Wavelet_LLL_glcm_homogeneity1	1	0.60	22	5	0.56	272
404	Wavelet_LLL_glcm_homogeneity2	1	0.60	23	5	0.56	271
405	Wavelet_LLL_glcm_infoCorr1	1	0.51	430	6	0.57	256
406	Wavelet_LLL_glcm_infoCorr2	4	0.54	390	4	0.53	335
407	Wavelet_LLL_glcm_invDiffmomnor	1	0.60	56	6	0.71	15
408	Wavelet_LLL_glcm_invDiffnorm	1	0.60	40	6	0.67	103
409	Wavelet_LLL_glcm_inverseVar	1	0.57	233	3	0.56	267
410	Wavelet_LLL_glcm_maxProb	5	0.59	96	5	0.53	353
411	Wavelet_LLL_glcm_sumAvg	1	0.56	321	1	0.66	126
412	Wavelet_LLL_glcm_sumEntro	4	0.58	177	4	0.53	359
413	Wavelet_LLL_glcm_sumSquares	1	0.56	312	1	0.66	121
414	Wavelet_LLL_glcm_sumVar	1	0.56	298	1	0.66	122
415	Wavelet_LLL_rlgl_grayLevelNonuniformity	6	0.61	13	7	0.68	66
416	Wavelet_LLL_rlgl_highGrayLevelRunEmphasis	1	0.56	292	1	0.66	118
417	Wavelet_LLL_rlgl_longRunEmphasis	5	0.60	28	5	0.61	197
418	Wavelet_LLL_rlgl_longRunHighGrayLevEmpha	5	0.59	62	1	0.67	99
419	Wavelet_LLL_rlgl_longRunLowGrayLevEmpha	2	0.51	433	8	0.63	166
420	Wavelet_LLL_rlgl_lowGrayLevelRunEmphasis	2	0.56	299	8	0.74	6
421	Wavelet_LLL_rlgl_runLengthNonuniformity	6	0.59	66	7	0.67	93
422	Wavelet_LLL_rlgl_runPercentage	4	0.60	24	9	0.60	200
423	Wavelet_LLL_rlgl_shortRunEmphasis	4	0.60	26	9	0.59	217
424	Wavelet_LLL_rlgl_shortRunHighGrayLevEmpha	1	0.53	408	1	0.64	148
425	Wavelet_LLL_rlgl_shortRunLowGrayLevEmpha	2	0.57	264	8	0.75	4
426	Wavelet_LLL_stats_energy	6	0.61	16	7	0.68	68
427	Wavelet_LLL_stats_entropy	4	0.59	118	4	0.50	426

428	Wavelet_LLL_stats_kurtosis	5	0.58	150	5	0.60	204
429	Wavelet_LLL_stats_max	7	0.58	170	2	0.66	114
430	Wavelet_LLL_stats_md	2	0.56	324	4	0.51	423
431	Wavelet_LLL_stats_mean	1	0.59	102	3	0.52	388
432	Wavelet_LLL_stats_median	1	0.61	9	3	0.52	383
433	Wavelet_LLL_stats_min	3	0.53	402	8	0.65	141
434	Wavelet_LLL_stats_range	7	0.57	222	2	0.67	106
435	Wavelet_LLL_stats_rms	1	0.59	77	3	0.54	325
436	Wavelet_LLL_stats_skewness	4	0.57	236	3	0.53	352
437	Wavelet_LLL_stats_std	2	0.54	392	4	0.56	277
438	Wavelet_LLL_stats_totalenergy	6	0.61	18	7	0.68	69
439	Wavelet_LLL_stats_uniformity	5	0.59	84	5	0.52	375
440	Wavelet_LLL_stats_var	2	0.54	393	4	0.56	276
